# Shielding assessment for multi-ion radiotherapy based on ion-specific dose-defined workloads

**DOI:** 10.1038/s41598-026-59143-0

**Published:** 2026-06-19

**Authors:** Ui-Seob Lee, Youngmoon Goh, Geum Mun Back, Chul Hee Min, Jinhong Jung, Jungwon Kwak, Si Yeol Song

**Affiliations:** 1 Medical Physics Technology Team, Heavy-ion Therapy Implementation Development, Asan Medical Center, Seoul, 05505 Republic of Korea; 2 Department of Radiation Convergence Engineering, Yonsei University, Wonju, Gangwon, 26493 Republic of Korea; 3 Department of Radiation Oncology, Asan Medical Center, University of Ulsan College of Medicine, Seoul, 05505 Republic of Korea

**Keywords:** Cancer, Oncology, Physics

## Abstract

The expansion of heavy-ion radiotherapy toward multi-ion operation requires a reassessment of shielding design methods that have traditionally relied on particle-number-based workloads. In facilities employing multiple ion species, this approach becomes inconsistent because different ions require substantially different numbers of primary particles to deliver the same prescribed dose. In this study, neutron shielding characteristics for helium, carbon, oxygen, and neon ion beams were evaluated using dose-defined workloads that reflect actual clinical and operational practice. Shielding effectiveness was assessed per unit physical dose to represent quality assurance and commissioning activities, and per unit Relative Biological Effectiveness (RBE)-weighted dose to represent treatment-related workload, based on PHITS Monte Carlo simulations. Under physical-dose normalization, carbon consistently produced the largest neutron effective dose across all shielding configurations. Under RBE-weighted dose normalization, carbon generally remained the most conservative reference ion, although high-energy helium under metallic beam-loss target conditions approached the carbon reference in selected configurations. These findings demonstrate that shielding outcomes are strongly dependent on the dose quantity used to define workload and support the continued use of carbon as the primary reference ion for multi-ion shielding assessment, while indicating that high-energy helium may approach the carbon reference only under restricted treatment-related metallic-target conditions.

**Supplementary Information:**

The online version contains supplementary material available at https://doi.org/10.1038/s41598-026-59143-0.

## Introduction

 As heavy-ion radiotherapy advances, clinical applications are expanding beyond conventional carbon-ion therapy to include additional ion species such as helium, oxygen, and neon. These ions exhibit distinct physical and biological characteristics that provide complementary therapeutic advantages, motivating the development of multi-ion therapy for more flexible and patient-specific treatment strategies, particularly for complex tumor geometries near radiosensitive organs^1–3^.

At the same time, the use of multiple ion species inevitably increases the complexity of facility operation and radiation protection, as beam delivery conditions, interaction mechanisms, and secondary radiation fields vary among ions^4,5^. Consequently, the expansion toward multi-ion operation necessitates a careful re-examination of shielding design principles that were largely developed for carbon-ion facilities.

In radiation shielding design, workload is intended to represent how a facility is actually operated and to quantify the cumulative production of secondary radiation relevant to long-term radiological safety. Accordingly, shielding calculations should be grounded in practical clinical and operational conditions rather than defined solely in terms of primary particle number. In practice, treatment delivery and quality assurance (QA) or commissioning activities are governed by different dose quantities, with clinical treatments prescribed in terms of Relative Biological Effectiveness (RBE)-weighted dose and QA procedures defined in terms of physical dose. In shielding design, calculations are commonly performed under conservative maximum-energy beam conditions, whether using Monte Carlo transport codes or analytical/semi-empirical approaches.

This distinction becomes particularly important in multi-ion therapy environments. Particle-number normalization is valid only when particle fluence and delivered dose are directly proportional; however, in multi-ion radiotherapy the particle-to-dose conversion differs substantially among ion species. Consequently, the number of primary particles required to deliver the same prescribed dose can vary substantially among ions. Under these conditions, particle-based workload definitions introduce an inherent inconsistency between clinical dose delivery and shielding assessment, potentially biasing comparisons of shielding burden and the identification of conservative reference conditions. Accordingly, shielding workload should be formulated on a dose basis, using physical dose for QA and commissioning activities and RBE-weighted dose for clinical treatments.

Previous studies have addressed shielding issues associated with advanced particle therapy systems. Dougherty et al. (2023)^6^ evaluated shielding for a next-generation synchrotron capable of delivering both proton and carbon-ion beams, demonstrating that carbon ions impose a substantially higher neutron burden than protons. Bonforte et al. (2023)^7^ extended such analyses to multiple ion species and proposed particle-based metrics to compare shielding burdens across ions. However, these studies relied primarily on normalization to the number of primary particles and did not account for differences in the particle numbers required to deliver the same clinically prescribed dose among ion species.

Dose-normalized descriptions have also been explored. Huang et al.^8^ characterized secondary neutron production in terms of ambient dose equivalent per therapeutic dose but focused exclusively on carbon-ion operation. As a result, it remains insufficiently addressed how shielding-relevant neutron quantities vary among different ion species under a common, operationally defined workload, particularly in the context of emerging multi-ion therapy facilities.

In this study, we evaluate neutron shielding performance in multi-ion therapy facilities using dose-defined workloads, rather than formulations based on primary particle number that have typically been applied in shielding analyses. For treatment-relevant comparisons, shielding effectiveness is assessed per unit RBE-weighted dose, representing the clinically prescribed quantity, while normalization to unit physical dose is also considered to reflect QA- and commissioning-related workloads. Using PHITS-based Monte Carlo simulations, we compare secondary neutron effective dose and related neutron field characteristics, including fluence and energy spectra, for helium, carbon, oxygen, and neon ion beams under these dose-based normalization schemes. This approach enables a clinically and operationally consistent comparison of shielding burdens across ion species and provides a robust basis for identifying conservative reference conditions in multi-ion therapy facilities.

## Methods

### Ion species and beam energies

Four ion species of clinical relevance, helium (He), carbon (C), oxygen (O), and neon (Ne), were selected for evaluation of secondary neutron production and shielding requirements. Beam energies were chosen to reflect clinically achievable or practically utilized conditions in heavy-ion radiotherapy. Carbon, oxygen, and neon ions were simulated at 430 MeV/u, corresponding to the maximum energies attainable in a synchrotron. Helium ions were evaluated at 220 MeV/u and 250 MeV/u, where 220 MeV/u provides a range comparable to that of 430 MeV/u carbon ions, and 250 MeV/u corresponds to the vendor-provided energy used for shielding calculations.

## Monte carlo simulation

Monte Carlo simulations were carried out with the Particle and Heavy Ion Transport code System (PHITS, version 3.35) using the JENDL-4.0 nuclear data library for cross-sectional information^9–11^. Nuclear interactions and fragmentation processes were treated with the built-in physics models of PHITS: The Intra-Nuclear Cascade of Liège (INCL) and JAM hadronic cascade models for nucleon-induced reactions, and the JAERI Quantum Molecular Dynamics (JQMD) model for heavy-ion collisions, applied according to projectile type and energy. The evaporation and fission processes were simulated using the Generalized Evaporation Model (GEM).

**Fig. 1:**
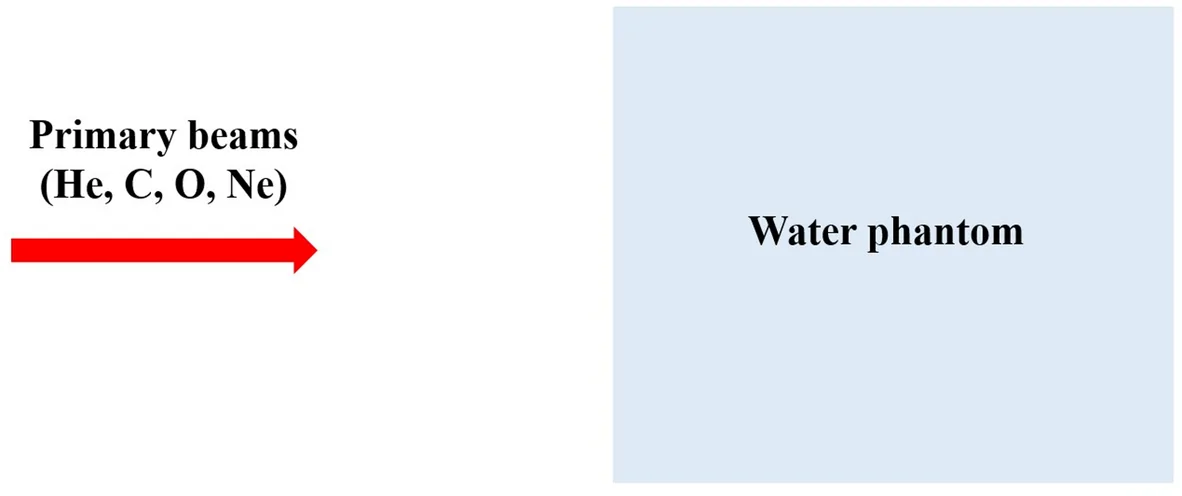
Simulation geometry for the pristine Bragg peak depth-dose calculation. Primary beams of He, C, O, and Ne were transported into a water phantom.

**Fig. 2:**
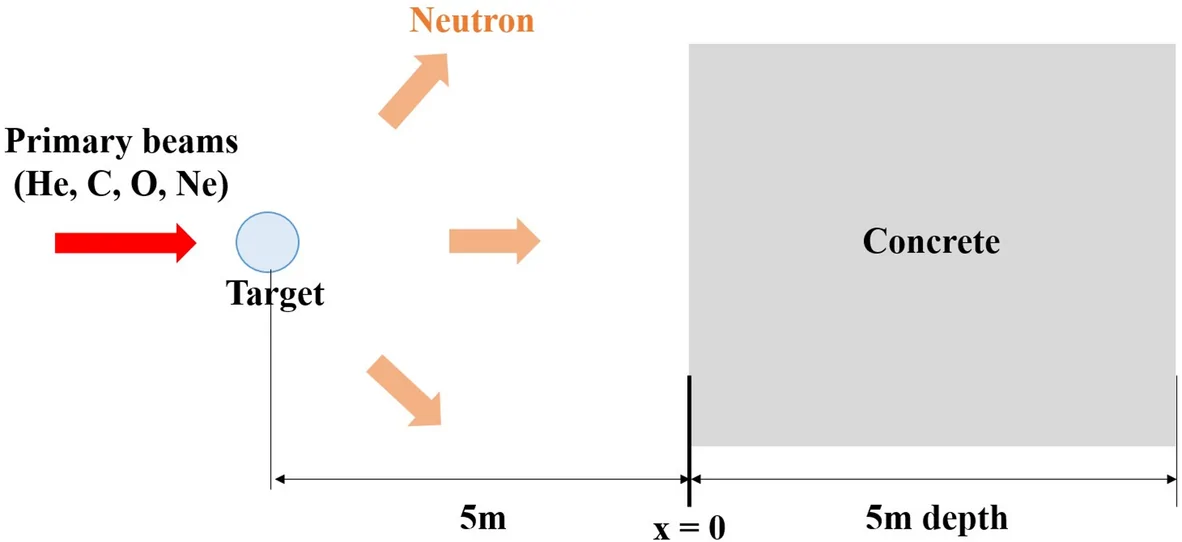
Simulation geometry for the shielding assessment. Primary beams of He, C, O, and Ne were incident on the target, and secondary neutrons were transported toward the concrete shielding wall. The target represents the beam-loss or reference medium considered in the shielding simulations, including water, copper, SUS304 electrostatic septum deflector (ESD), and silicon steel, as specified in Table 1. Depth x in the shielding wall was calculated from the upstream surface of the concrete wall, defined as x = 0.

Figure 1 illustrates the simulation configuration used to obtain pristine Bragg peaks, and Fig. 2 illustrates the shielding assessment geometry. Table 1 summarizes the material compositions used in the simulations. For each ion species, the incident beam was modeled as a monoenergetic point source without initial angular divergence.

**Table 1:** Material densities and elemental compositions used in the simulations.

Material and modeled component	Density (g/cm^3^)	Elemental composition (atomic fraction, %)
Water (Isocenter)	1.0	H (66.7), O (33.3)
Copper (Beam stopper/dumper)	8.9	Cu (100)
SUS304 Electrostatic septum deflector (ESD)	7.9	C (0.4), Si (1.9), P (0.1), S (0.1), Cr (20.0), Mn (2.0), Fe (66.3), Ni (9.3)
Silicon steel (Bending magnet)	7.8	Si (7.7), Fe (92.3)
Concrete (Shielding wall)	2.2	H (17.1), C (1.3), O (56.0), Al (2.1), Si (20.5), Ca (2.6), Fe (0.4)

In the water-phantom scenario, a homogeneous water phantom was placed directly in the beam path to obtain depth-dose distributions (pristine Bragg peaks) for all ion species. Microdosimetric lineal-energy distributions, d(y), were also tallied to derive depth-dependent lineal-energy spectra for subsequent RBE analysis. The pristine Bragg peak calculations were performed for the maximum-energy conditions used in the shielding assessment, including 430 MeV/u for carbon, oxygen, and neon and 220 and 250 MeV/u for helium.

In addition, an SOBP-based analysis was performed in the water phantom to account for clinically extended dose delivery in the dose-based workload normalization. For each ion, SOBPs with widths of 1, 3, and 5 cm were constructed with the distal edge placed at the maximum range corresponding to the maximum beam energy used in the shielding assessment. The SOBPs were generated by combining Monte Carlo-calculated pristine Bragg peak depth-dose distributions with energy-dependent weighting factors to obtain flat RBE-weighted dose distributions over the SOBP region. The RBE-weighted dose was then integrated over each SOBP region, and the corresponding ion-specific particle-number-per-dose factors were recalculated. This SOBP analysis was applied only to the water-phantom dose-normalization step.

In the shielding assessment scenario, the neutron transport calculations were performed using the maximum-energy pristine beams as reference source conditions. This was done because the most penetrating secondary neutrons relevant to conservative external shielding assessment are predominantly produced by the highest-energy beam component. Four representative materials corresponding to major beam-loss locations in a heavy-ion therapy facility were modeled: the beam stopper/dumper, the electrostatic septum deflector (ESD), bending magnets, and the isocenter. For the beam stopper/dumper, where the primary beam directly interacts with copper, a 10-cm-thick copper target was placed 5 m upstream of a homogeneous concrete shielding wall with a thickness of 5 m. For the electrostatic septum deflector (ESD) and the bending magnets, beam losses were modeled using SUS304 and silicon steel targets, respectively, positioned at the same upstream location. For the isocenter, where the primary beam interacts with tissue-equivalent material, a 40-cm-thick water target was placed 5 m upstream of the shielding wall.

Across all configurations, shielding was modeled using a concrete barrier with a total thickness of 5 m in order to capture the full attenuation behavior of secondary neutrons and to derive tenth-value layers (TVLs), defined as the additional concrete thickness required to reduce the neutron dose equivalent by one order of magnitude. While the full barrier thickness was extended beyond typical clinical dimensions for this purpose, representative inter-ion comparisons are reported at a depth of 200 cm, corresponding to a typical primary-barrier thickness in clinical facilities. To evaluate the influence of dense, high-Z shielding reinforcement on neutron attenuation, an additional composite shielding configuration was modeled by placing a 50-cm iron layer in front of the concrete barrier. The iron layer was explicitly included in the PHITS geometry to account for both high-energy neutron attenuation and secondary neutron production within the high-Z material. This configuration was intended to represent a practical local reinforcement strategy applicable to heavy-ion shielding design. The results for this composite shielding configuration are presented in the Supplementary Information (Supplementary Figure S1).

Neutron effective doses were calculated using the fluence-to-effective-dose conversion coefficients from ICRP Publication 116^12^ for the anterior-posterior (AP) irradiation geometry, based on the ICRP reference adult phantom. The AP geometry was selected as a conservative approximation for external exposure. All neutron dose results were subsequently scaled to an equivalent physical dose or RBE-weighted dose across ion species. Angularly and energetically resolved neutron spectra were extracted at 10° intervals using a spherical air scoring volume surrounding the target, enabling full angular characterization of secondary neutron emissions.

## Dose-based shielding assessment

In this study, shielding effectiveness was evaluated on the basis of dose-defined workloads, rather than primary-particle fluence-based metrics. Treatment-related shielding was evaluated per unit RBE-weighted dose, consistent with clinical dose prescription, whereas shielding relevant to quality assurance (QA) and commissioning was evaluated per unit physical dose. Under both evaluation schemes, the number of incident particles was treated as a derived quantity determined by the required dose and beam characteristics of each ion species, rather than as an independent operational parameter.

For dose-based shielding assessment, the workload was defined by integrating the relevant dose quantity along depth in water. For each ion, we located the distal half-maximum crossing ($\:{z}_{R50}$) on the physical dose or RBE-weighted dose profile and define an integration window of width L extending proximally from this point. In the pristine-peak analysis, the integration length L was used as a reference scoring interval for comparing dose per primary particle among ion species under controlled conditions. We computed the dose integral

1 $$\:A\left(L\right)={\int\:}_{{Z}_{R50}-L}^{{Z}_{R50}}{D}_{RBE\:or\:phys}\left(z\right)dz.$$

using numerical integration methods, Simpson’s rule, and defined the inverse-area metric (I)

2 $$\:I\left(L\right)=\:\frac{L}{A\left(L\right)}\:\:\:\left[\frac{particle\:\#}{Gy\:or\:Gy\left(E\right)}\right].$$

which is proportional to the number of particles required to deliver the same physical dose or RBE-weighted dose in the integration window. To test the dependence of the dose-based workload on the integration length L around $\:{z}_{R50}$ in water, we repeated the normalization using window lengths between 1 and 6 cm and recalculated the required number of primary particles per unit RBE-weighted dose. To evaluate clinically extended target-dose delivery, an additional SOBP-based analysis was performed. In this analysis, the RBE-weighted dose was integrated over the SOBP region, and the corresponding ion-specific particle-number-per-dose factors were calculated.

The effective dose per primary particle, obtained by multiplying the simulated neutron fluence per primary ion by the ICRP 116 conversion coefficient, was then multiplied by the ion-specific particle-number-per-dose factor, yielding the effective neutron dose per unit physical dose [pSv/Gy] or per unit RBE-weighted dose [pSv/Gy(E)]. This dose-based rescaling ensures that all ions are compared under equivalent dose delivery, allowing shielding performance to be assessed on a clinically meaningful basis.

## RBE-weighted dose from modified Microdosimetric Kinetic Model (MKM)

For treatment-relevant shielding comparisons based on RBE-weighted dose, the physical dose distributions obtained from PHITS were converted to RBE-weighted dose using the modified microdosimetric kinetic model (MKM)^13–16^. The modified MKM was selected because it is widely used in scanned carbon-ion clinical dose systems and explicitly accounts for RBE saturation (overkill) effects in the high-LET region, which is relevant around the Bragg peak. Using a single, internally consistent RBE model across ion species avoids confounding inter-ion comparisons by differences in model formulation. The dose probability distribution of lineal energy, d(y), was used to calculate the saturation-corrected dose-mean specific energy $\:{z}_{1D}^{*}$, according to

3 $$\:{z}_{1D}^{*}\:=\:\frac{1}{\rho\:\pi\:{r}_{d}^{2}}{y}_{0}^{2}{\int\:}_{0}^{\infty\:}\frac{1-\mathrm{e}\mathrm{x}\mathrm{p}(-{y}^{2}/{y}_{0}^{2})}{y}d\left(y\right)dy.$$

where $\:\rho\:$ is the density, $\:{r}_{d}$ is the radius of the domain, and $\:{\mathrm{y}}_{\mathrm{0}}$ is the saturation parameter (108 keV/µm). The resulting $\:{\mathrm{z}}_{\mathrm{1D}}^{\mathrm{*}}$ then enters the LQ formalism:

4 $$\:\alpha\:\left(z\right)={\alpha\:}_{0}+\beta\:{z}_{1D}^{*}\left(z\right)$$

with $\:{\alpha}_{\mathrm{0}}$= 0.155 Gy^−1^ and $\:\beta\:$ was fixed to 0.0615 Gy^−2^^15^. These fixed $\:{\alpha}_{\mathrm{0}}$ and $\:\beta\:$ values were used because the clinical modified MKM-based RBE dose convention adopted in carbon-ion therapy is defined using fixed reference cell-survival parameters. From these parameters, we calculated the 10% survival dose ($\:{D}_{10}$) from the linear-quadratic model, and obtained the corresponding RBE, referenced to a 200 kVp X-ray.

5 $$\:{RBE}_{10}=\:\frac{{D}_{10,\gamma\:}}{{D}_{10}}$$

Finally, the RBE-weighted dose is obtained by

6 $$\:{D}_{RBE}=\:{RBE}_{10}\:\times\:\:{D}_{phys}.$$

where D_phys_ is the physical dose in the water-phantom obtained from PHITS. All model constants were referenced from HSG cell-survival data^13–15^.

### Statistical analysis

Statistical uncertainties were quantified using the PHITS-reported relative errors, corresponding to one-sigma (68% confidence level) standard deviations of the Monte Carlo tallies. These values represent the stochastic fluctuations inherent to the particle transport process and do not account for systematic uncertainties associated with the physics models or nuclear data. For tallies based on accumulated energy deposition, the statistical uncertainty scales approximately with the inverse square root of the number of contributing particle histories, reflecting the convergence behavior of Monte Carlo simulations.

Under these simulation conditions, a convergence criterion was imposed such that all tallies were required to reach a PHITS‑reported relative error below 0.05, ensuring that the one‑sigma statistical uncertainty remained below 5%. The reported statistical uncertainties represent Monte Carlo tally uncertainties only. They do not include model-related systematic uncertainties arising from nuclear reaction models, evaluated nuclear data libraries, de-excitation models, or neutron transport in shielding materials. For comparisons expressed as ratios between two independently tallied quantities (e.g., He/C), the one-sigma uncertainty of the ratio was estimated using first-order error propagation.

## Results

### Physical dose and RBE-weighted dose distributions

**Fig. 3:**
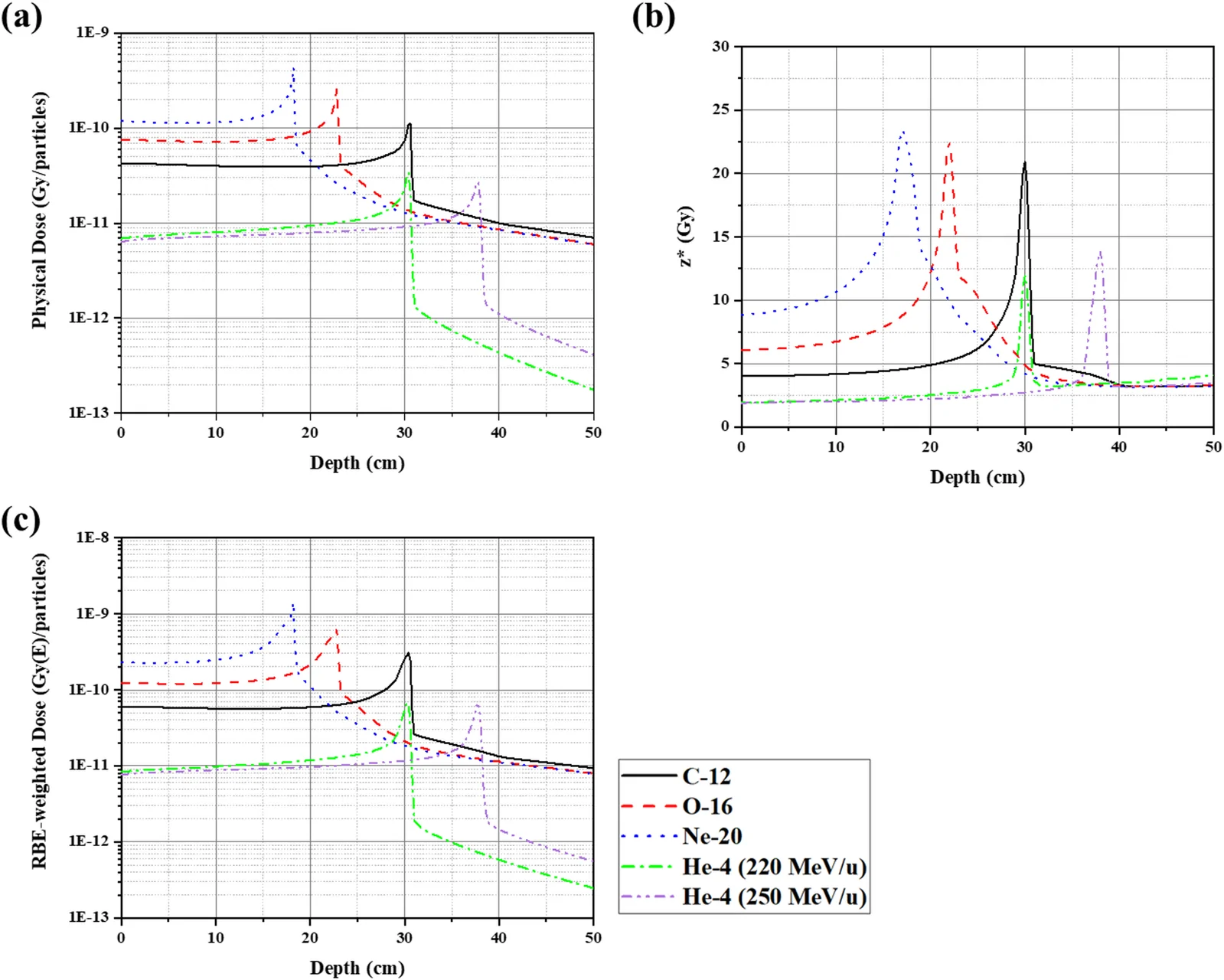
(**a**) Pristine-peak physical dose distributions in water for helium, carbon, oxygen, and neon ion beams, (**b**) Saturation-corrected dose-mean specific energy $\:{z}_{1D}^{*}$ for each ion, and (**c**) Depth-dependent RBE-weighted dose distributions obtained by applying modified MKM based RBE factors to the corresponding physical dose profiles.

Figure 3(a) presents the pristine-peak physical dose distributions in water for carbon, oxygen, neon, and helium ions. Carbon ions delivered at 430 MeV/u and helium beams at 220 MeV/u exhibited a Bragg peak at a depth of 30.6 cm. Helium beams delivered at 250 MeV/u penetrated to a greater depth of approximately 38.0 cm, whereas oxygen and neon beams delivered at the same maximum energy per nucleon (430 MeV/u) stopped at shallower depths of approximately 23.0 cm and 18.4 cm, respectively, reflecting the reduced penetration range of heavier ions at fixed energy per nucleon. The peak physical dose per primary ion increased with ion mass. Relative to carbon, the peak physical doses of oxygen and neon were approximately 2.4 and 4.0 times higher, respectively, indicating increasingly concentrated end-of-range energy deposition for heavier ions. In contrast, the peak physical dose of helium at 250 MeV/u was lower than that of carbon, amounting to approximately 0.27 of the carbon value. For comparison, helium at 220 MeV/u exhibited a slightly higher peak dose per primary than the 250 MeV/u beam, corresponding to approximately 1.25 times the 250 MeV/u value.

Figure 3(b) shows the depth-dependent saturation-corrected dose-mean specific energy $\:{z}_{1D}^{*}$. The peak value for helium at 250 MeV/u was approximately 0.66 times that for carbon, and the peak for 220 MeV/u helium was lower, at about 0.57 of the carbon value. In comparison, the peaks for oxygen and neon were 1.08 and 1.12 times the carbon value, respectively, indicating a monotonic increase in the local Linear Energy Transfer (LET) from helium to neon. Beyond the peak region, distinct differences were observed in the distal tail of the $\:{z}_{1D}^{*}$ distributions. For carbon ions, $\:{z}_{1D}^{*}$ decreased gradually with depth, remaining at approximately one quarter of the peak in the distal region. As the ion mass increased, the magnitude of the high-specific-energy tail became more pronounced: for oxygen and neon, the distal tail exhibited $\:{z}_{1D}^{*}$ values approximately 55% of the peak value, followed by a gradual decrease with depth. In contrast, helium exhibited substantially lower tail values, with $\:{z}_{1D}^{*}$ in the distal region remaining comparable to those in the plateau region and showing no pronounced enhancement beyond the Bragg peak.

Figure 3(c) presents the depth-dependent RBE-weighted dose distributions obtained by applying RBE factors derived from the microdosimetric kinetic model to the corresponding physical dose profiles. Among the ions considered, neon exhibited the highest peak RBE-weighted dose per primary, approximately 4.7 times that of carbon and about 2.1 times that of oxygen. Helium exhibited substantially lower peak RBE-weighted dose than carbon. The peak values for helium at 220 and 250 MeV/u were very similar, both on the order of approximately 0.22 of the carbon reference, indicating only a weak dependence on beam energy within this range.

## Particle number normalization for dose-based workload calculation

We computed particle number ratios for helium, oxygen, and neon required to deliver the same physical dose and the same RBE-weighted dose. To ensure consistent normalization across ion species, the dose was integrated over a depth interval anchored at the distal half-maximum position, denoted as $\:{z}_{R50}$. An integration window length of 2.0 cm was adopted, as this window fully encompassed the half-maximum region for all ions considered.

When normalization was based on RBE-weighted dose, representing treatment-related workload, helium required substantially more primary particles than carbon to achieve the same dose. For an integration window length of 2.0 cm, helium delivered at 250 MeV/u required approximately 5.63 times the number of primary particles used for carbon, while helium at 220 MeV/u required about 5.25 times. By contrast, oxygen and neon achieved the same RBE-weighted dose with fewer primary particles than carbon, with ratios of approximately 0.46 and 0.26, respectively.

Applying the same integration procedure with physical dose as the normalization quantity, relevant to QA- and commissioning-related workload, resulted in similar inter-ion behavior, with different absolute ratio values. Under physical-dose normalization, helium at 220 MeV/u required roughly 3.5 times the number of primary particles needed for carbon, and this factor increased to approximately 4.0 for helium at 250 MeV/u. Oxygen and neon again exhibited lower particle-number requirements than carbon, with corresponding ratios of approximately 0.52 and 0.33, respectively.

To assess sensitivity to the choice of integration length, the normalization was repeated using window lengths ranging from 1 to 6 cm around $\:{z}_{R50}$. While larger variations were observed for short integration windows that partially captured the peak region, the particle-number ratios remained stable within approximately 5% for window lengths of 2.0 cm and larger.

Under SOBP-based normalization, the particle-number ratios relative to carbon were approximately 5.07 for helium, 0.48 for oxygen, and 0.30 for neon. These ratios were nearly unchanged across SOBP widths of 1, 3, and 5 cm, indicating that the modulation width had little effect on the dose-based particle-number normalization within the range examined.

As an additional check, when the SOBP-based particle-number normalization was applied to the RBE-weighted-dose-normalized shielding burden, the helium-to-carbon normalization factor decreased from 5.63 for the pristine-peak normalization to approximately 5.07 for the SOBP-based normalization. This corresponds to an approximately 10% relative reduction in the helium-normalized shielding burden compared with the pristine-peak-based estimate. For oxygen and neon, the SOBP-based particle-number ratios were approximately 0.48 and 0.30, respectively, which were also lower than the corresponding pristine-peak-based ratios of 0.52 and 0.33. These results indicate that the use of SOBP-based clinically extended dose normalization does not reduce the conservativeness of the carbon-based shielding assessment under the conditions examined in this study.

### Depth-dependent neutron effective dose across ion species and shielding structures

**Fig. 4:**
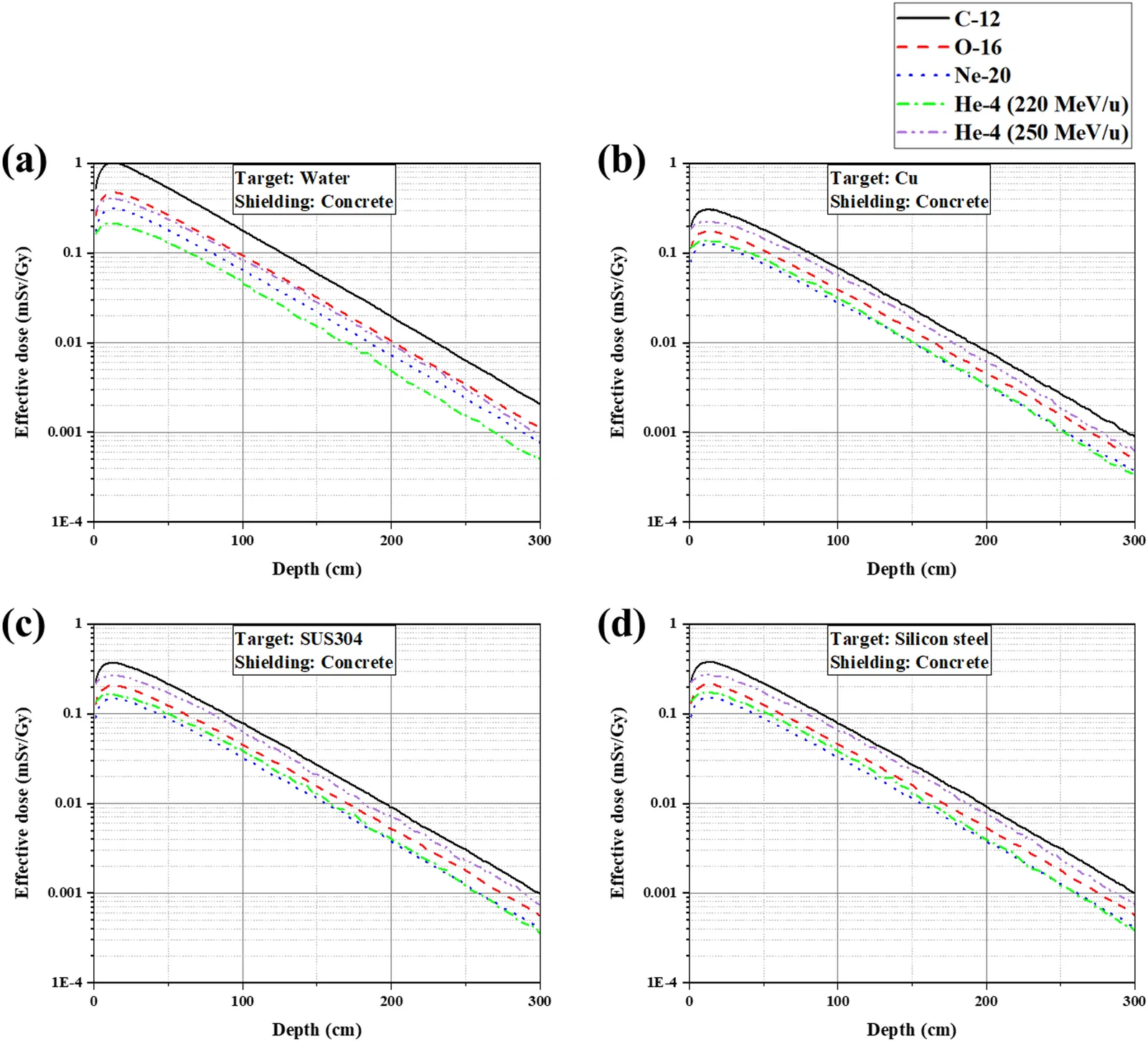
Depth-dependent neutron effective dose per unit physical dose for helium, carbon, oxygen, and neon beams across (**a**) water, (**b**) copper, (**c**) SUS304, and (**d**) Silicon steel target and concrete shielding configurations.

Figure 4 shows depth-dependent neutron effective doses for helium, carbon, oxygen, and neon ions after scaling by physical dose, representing the shielding burden per unit physical dose relevant to QA- and commissioning-related workload. Across all investigated shielding configurations, including H₂O–concrete, Cu–concrete, SUS304–concrete, and silicon steel–concrete geometries, and throughout the full depth range, carbon consistently produced the largest physical dose-normalized neutron effective dose. For all conditions considered, the contributions from helium, oxygen, and neon remained below the carbon reference, although their relative magnitudes depended on ion species and beam energy. The addition of an iron layer in front of the concrete wall uniformly reduced the absolute neutron effective doses at the concrete boundary, without altering the relative trends among ion species (Supplementary Figure S2 and S3).

**Fig. 5:**
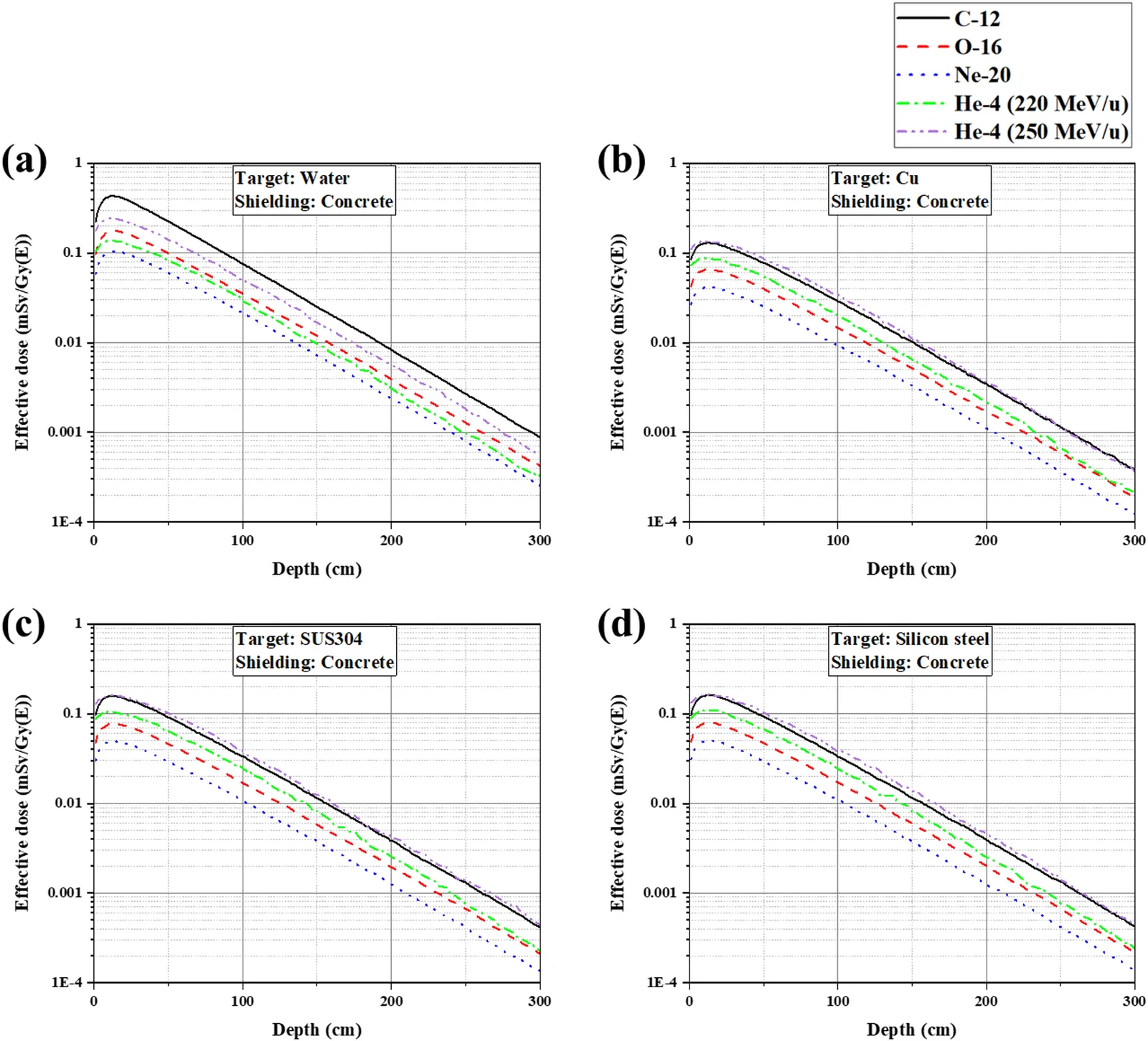
Depth-dependent neutron effective dose per unit RBE-weighted dose for helium, carbon, oxygen, and neon beams across (a) water target, (b) copper target, (c) SUS304 target, and (d) Silicon steel target and concrete shielding configurations.

Figure 5 presents the neutron effective doses normalized to RBE-weighted dose, representing treatment-related workload. Across most investigated configurations and depths, carbon exhibited the highest RBE-weighted neutron effective dose. For configurations involving water targets, including both H₂O–concrete and H₂O–Fe–concrete geometries, carbon consistently produced the largest normalized dose across depths, followed by helium at 250 MeV/u, oxygen, helium at 220 MeV/u, and neon. However, for metallic beam-loss materials, distinct behavior was observed for high-energy helium. Under the Cu–concrete configuration, helium at 250 MeV/u produced slightly higher neutron effective doses than carbon in the proximal shielding region; for example, at a depth of 200 cm (representative of a typical primary-barrier thickness), the normalized dose for helium at 250 MeV/u was approximately 4.8% higher than that of carbon. For this condition, the PHITS-reported relative errors were 0.038 for helium and 0.016 for carbon, corresponding to an estimated one-sigma uncertainty of approximately 4.1% on the He/C ratio. The observed excess is therefore marginal even when considering statistical uncertainty alone, and should be interpreted as comparable within the broader model-related uncertainty rather than as evidence of a higher helium shielding burden.

Similar trends were observed for iron-dominated materials. At a depth of 200 cm, helium at 250 MeV/u yielded neutron effective doses approximately 9.7% (with a relative uncertainty of 3.7%, 1σ) higher than carbon for the SUS304–concrete configuration and approximately 14.6% (with a relative uncertainty of 3.9%, 1σ) higher than carbon for the silicon steel–concrete configuration. In contrast, oxygen, helium at 220 MeV/u, and neon yielded clearly lower normalized doses than carbon over the full depth range for these materials. These results indicate that, when RBE-weighted dose is considered, the ion species associated with the highest neutron effective dose can depend on both beam energy and the composition of the beam-loss material.

As an additional shielding indicator, the tenth-value layers averaged across all medium compositions were approximately 97 cm for helium and 101–102 cm for carbon, oxygen, and neon. The addition of an iron layer did not alter the equilibrium-region TVLs, defined as the TVLs evaluated from the approximately exponential attenuation region in the downstream concrete, after excluding the local dose enhancement caused by the iron layer and the dose variation near the material transition.

### Energy spectrum of scattered neutrons

A preceding analysis based on normalization to unit RBE-weighted dose indicated a tendency for He-4 at 250 MeV/u to produce a higher neutron effective dose than C-12 under selected material conditions. To further explore the physical basis of this behavior, the present section examines (i) the dose-scaled forward neutron yield and (ii) the energy composition of forward-scattered neutrons, using scattering-angle resolved neutron fluence and forward-cone spectral analysis.

Neutron fluence generated in each target material was partitioned into 10° scattering-angle bins, and the forward component was quantified for neutrons scattered into the forward hemisphere (≤ 90°). For a given target material, the forward fraction varied only weakly among the incident ions, whereas a pronounced difference was observed between materials. In water, approximately 89% of the scattered neutrons were emitted into the forward hemisphere for all ion species, while in copper the corresponding fraction decreased to about 66%, indicating enhanced angular broadening relative to water. For the other metallic beam-loss targets, the forward fractions were similarly reduced, averaging approximately 70.3% for SUS304 and 71% for silicon steel, confirming a common material-dependent broadening effect across the metallic media considered.

**Fig. 6:**
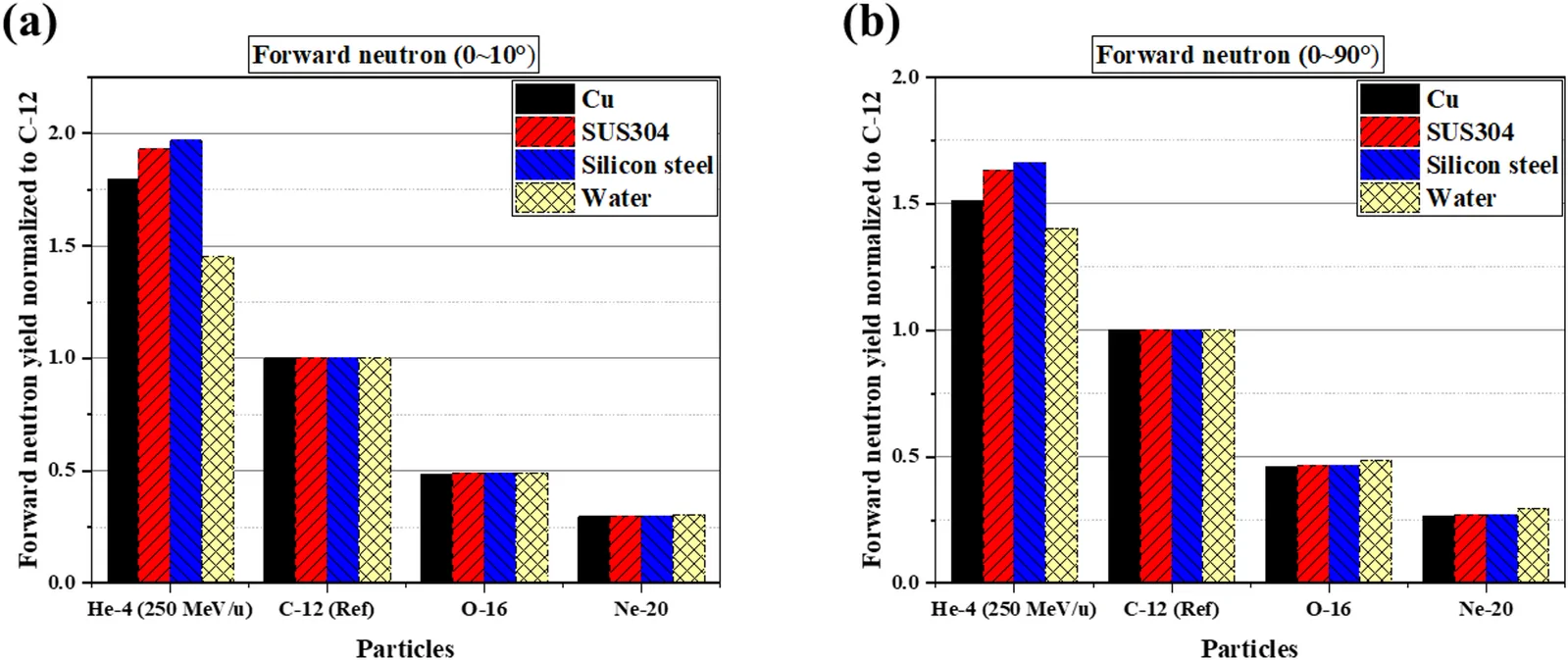
(**a**) Dose-scaled forward neutron yields for neutrons scattered within 0–10° and (**b**) dose-scaled forward neutron yields integrated over the forward hemisphere (0–90°) for He-4 (250 MeV/u), C-12, O-16, and Ne-20 interacting with water, Cu, SUS304, and silicon steel targets. For each target material, neutron yields are normalized to carbon (C-12). Yields are scaled by the number of primary particles required to deliver an equal dose for each ion species, enabling direct comparison of shielding-relevant neutron production under dose-defined workloads.

Figure 6 summarizes the dose-scaled forward neutron yields, normalized to carbon (C-12) for each target material. After scaling by the number of primary particles required to deliver an equal RBE-weighted dose, helium exhibited the largest material dependence among the ions considered. The forward yield of He-4 (250 MeV/u) in water was 45% higher than that of carbon, whereas the corresponding excess increased to 80% in Cu, 93% in SUS304, and 97% in silicon steel, indicating a markedly stronger relative contribution of helium in metallic beam-loss targets than in water. By contrast, oxygen and neon exhibited little dependence on the metallic beam-loss target material: their carbon-normalized forward yields remained nearly unchanged across Cu, SUS304, and silicon steel (within 2%). In water, however, these yields increased relative to the metallic beam-loss target values.

**Fig. 7:**
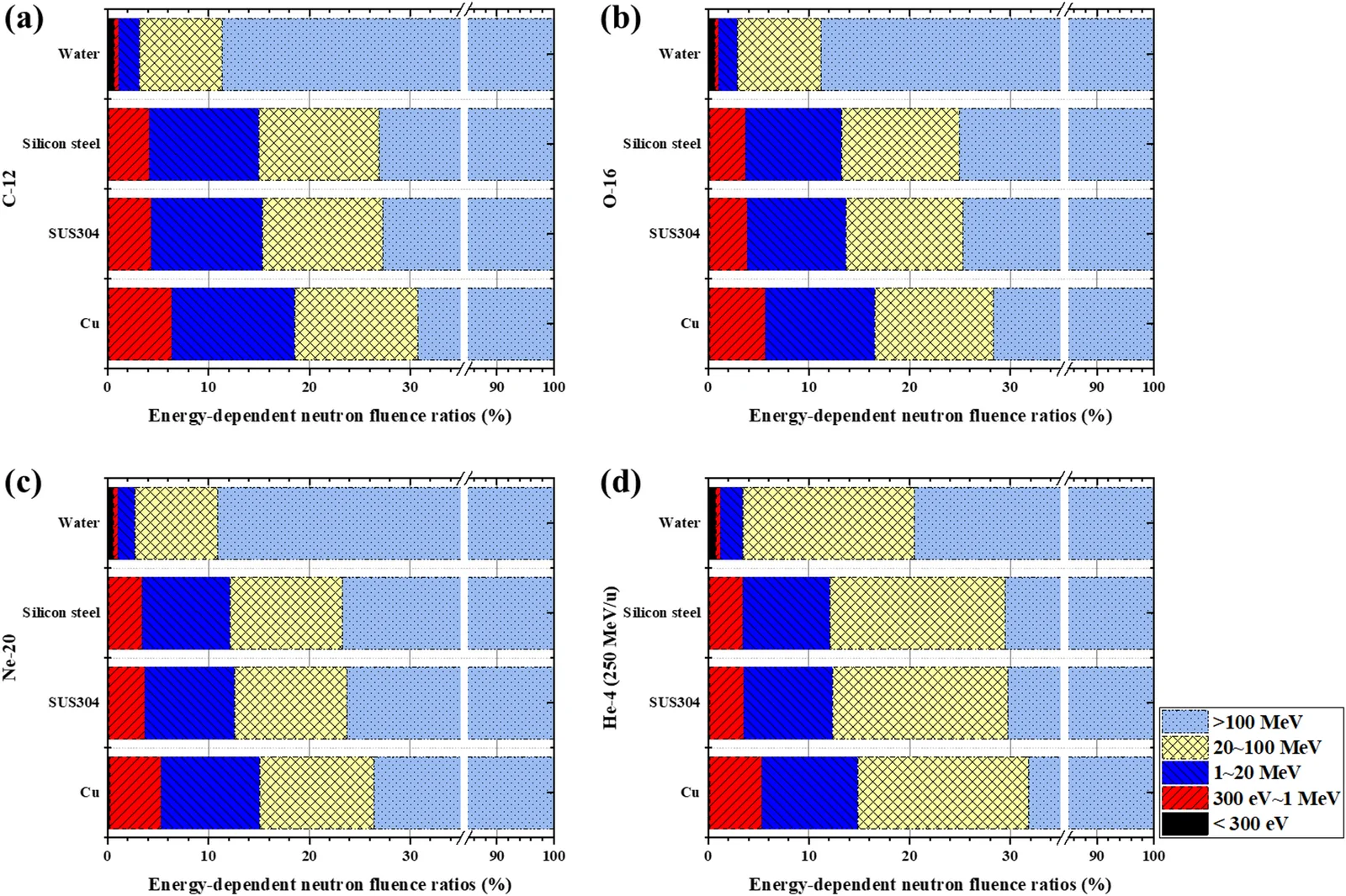
Energy-dependent neutron fluence ratios of forward-scattered neutrons within 0–10° for (**a**) C-12, (**b**) O-16, (**c**) Ne-20, and (**d**) He-4 (250 MeV/u) interacting with water, Cu, SUS304, and silicon steel targets. For each ion species, neutron fluence fractions are shown as a function of target material and grouped into five energy ranges: <300 eV, 300 eV–1 MeV, 1–20 MeV, 20–100 MeV, and > 100 MeV. The distributions highlight material-dependent changes in the forward-cone energy composition while demonstrating that, for metallic beam-loss targets, the relative spectral structure remains similar across ion species. The x-axis is broken to improve the visibility of the lower- and intermediate-energy components; all bars represent 100% of the forward-cone neutron fluence.

The spectral basis of this behavior was evaluated using the neutron energy distributions within the forward cone (0–10°), grouped into standard energy ranges; <300 eV, 300 eV–1 MeV, 1–20 MeV, 20–100 MeV, and > 100 MeV. Figure 7 presents the corresponding energy-dependent neutron fluence fractions. In water, the forward-scattered neutron fluence was dominated by neutrons above 100 MeV, accounting for nearly 90% of the forward component for carbon, oxygen, and neon, but only about 79% for helium. In copper, the fraction above 100 MeV decreased for all ions to approximately 68–74%, and the absolute difference in this high-energy fraction between helium and the heavier ions was reduced to about 1–6%; SUS304 and silicon steel exhibited comparable forward-cone spectral structures to copper, with similarly narrowed inter-ion differences in the high-energy fraction. Taken together, these observations indicate that metallic beam-loss targets shift the forward-cone spectrum toward lower energies and broader angles relative to water, while maintaining broadly similar spectral shapes among ions.

## Discussion

### Dose-based evaluation and conservative reference selection for multi-ion shielding

This study builds on the premise that shielding workload should represent how a heavy-ion therapy facility is actually operated, rather than serving as a purely computational convenience. In conventional single-ion facilities, normalization by primary particle number remains a reasonable approximation because the relationship between particle fluence and delivered clinical dose is effectively fixed. In multi-ion therapy environments, however, this assumption no longer holds. Different ion species exhibit markedly different stopping powers, fragmentation behavior, and biological effectiveness, leading to substantial differences in the number of primary particles required to deliver the same prescribed dose. As a result, normalization based on particle number is not an appropriate basis for inter-ion shielding comparisons, as it fails to represent dose-defined shielding workload.

An additional operational consideration arises from the distinction between treatment delivery and quality assurance (QA) or commissioning activities. In clinical practice, treatment beams are prescribed and evaluated in terms of RBE-weighted dose, whereas QA and commissioning procedures are defined and documented primarily in terms of physical dose. A single particle-based workload definition therefore cannot simultaneously represent these two operational regimes. Defining shielding workload in terms of dose quantities that reflect actual clinical and operational practice provides a more consistent basis for evaluating shielding performance in multi-ion facilities.

Within this dose-defined formulation, each ion species was evaluated under the assumption that it accounts for the full treatment or QA workload, without imposing speculative assumptions regarding relative clinical usage. This approach allows intrinsic inter-ion differences in shielding performance to be examined independently of hypothetical usage scenarios and avoids ambiguity in identifying conservative reference conditions.

When shielding performance is evaluated on a per-primary basis, heavier ions such as oxygen and neon exhibit higher neutron yields per incident ion, consistent with their larger nuclear interaction cross sections and higher fragmentation probability. Under these conditions, neon produces the largest neutron effective dose outside the shielding among the ions considered, followed by oxygen and carbon. This trend is particularly pronounced in configurations involving high-Z materials, where forward-directed high-energy neutrons contribute significantly to the external dose. When evaluated on a per-particle basis, neon appears to be the most conservative reference ion because of its high neutron yield per primary ion; under the copper condition at 200 cm, for example, the effective dose from neon was approximately 23% higher than that from carbon, consistent with the 25.8% higher neutron ambient-dose equivalent reported by Agosteo et al. (2004)^17^.

However, shielding evaluation based on physical-dose normalization yields inter-ion trends that differ fundamentally from those obtained on a per-primary basis. Under physical-dose normalization, oxygen and neon required substantially fewer primary particles than carbon to deliver the same dose, approximately 52% and 33% of the carbon particle number, respectively, and consequently exhibited lower neutron effective doses on a per-dose basis. Helium, by contrast, required a markedly larger number of primary particles to achieve the same physical dose, about 3.5 times for 220 MeV/u and 4.0 times for 250 MeV/u relative to carbon. Notably, the neutron effective dose per primary ion for helium was significantly lower than that for carbon, reduced by about 80 to 85%. As a result, even after accounting for the increased particle number, the neutron effective dose normalized to physical dose for helium remained below the carbon reference.

Additional distinctions emerge when RBE-weighted dose is adopted as the normalization quantity, representing treatment-related workload. Under RBE-weighted normalization, carbon generally produces the highest neutron effective dose across shielding configurations. Under restricted combinations of high helium beam energy and metallic beam-loss target materials (Cu, SUS304, and silicon steel), however, helium at 250 MeV/u locally approaches or marginally exceeds the carbon-based contribution, representing a conditional reversal of the inter-ion relationship. This behavior reflects the increased sensitivity of helium to beam energy and material composition when a larger number of primary particles is required to deliver the same RBE-weighted dose.

Taken together, these results demonstrate that conclusions regarding conservative shielding conditions depend explicitly on the choice of dose normalization. Under most clinically relevant conditions, carbon-ion operation provides a robust conservative reference, indicating that shielding designs derived from carbon-ion workloads remain adequate for multi-ion facilities. Retaining carbon as the reference species allows existing shielding designs that have already been validated and approved in operational facilities to remain the basis of assessment, thereby minimizing unnecessary cost and large-scale structural modification.

The observation that high-energy helium may locally exceed carbon under specific conditions is intended as a conservative boundary check rather than as evidence of a systematic dominance of helium. This finding should not be interpreted as establishing helium as a generally more conservative reference ion or as warranting a revision of carbon-based shielding criteria. Instead, it serves to confirm the robustness of carbon-based shielding designs while identifying limited scenarios in which additional verification may be warranted. Therefore, helium-ion operation should be treated as a conditional case requiring focused verification under specific treatment-related conditions, rather than as a basis for redefining the overall shielding design.

The present analysis used ICRP 116 fluence-to-effective-dose conversion coefficients for the AP irradiation geometry, which was selected as a conservative directional conversion condition for the primary-barrier shielding assessment considered here. In actual treatment rooms, ISO or ROT geometries may be more appropriate for locations dominated by room-scattered, rotational, or maze-like radiation fields. Therefore, we additionally compared AP- and ISO-based effective doses for the representative Cu–concrete configuration. The ISO-based values were approximately 20% lower than the AP-based values across the evaluated shielding-depth range (Supplementary Table S1). These results indicate that the principal ion-dependent shielding trends and conservative reference selection are not materially altered by the use of AP conversion coefficients.

### Interpretation based on ion-specific nuclear interactions

To provide additional insight into the physical basis of the inter-ion differences observed under dose-based normalization, we examined the dose-scaled forward neutron yield (Fig. 6) together with the angular and spectral characteristics of the scattered neutrons (Fig. 7). Overall, the angular–energy characteristics were governed primarily by the target material, whereas the projectile species mainly modulated the absolute magnitude of neutron production.

In water, forward emission remains pronounced for all ions and the forward 0–10° spectra retain a substantial high-energy component. Heavier ions (carbon, oxygen, and neon) exhibit a markedly larger fraction of neutrons above 100 MeV than helium. In copper, by contrast, the forward fraction decreases for all ions and the fraction above 100 MeV contracts across species, with a larger absolute reduction observed for the heavier ions than for helium ions. Similar behavior is also observed for the SUS304 and silicon steel targets, whose forward-cone (0–10°) energy spectra were comparable to those of copper. This strong material dependence narrows the inter-ion species differences in the forward-cone high-energy component, reducing the contrast in the fraction of neutrons above 100 MeV when transitioning from water to metallic beam-loss targets.

As a source-term assessment, the angular-energy neutron fluence spectra for the 430 MeV/u carbon-on-water condition were converted to double-differential neutron yields using the T-cross scoring geometry. This condition was selected because it provides the most directly corresponding experimental reference for the carbon reference ion and the patient-like water target considered in this study. The converted spectra were shown in the same plotting format as the water-phantom measurements reported by Satoh et al.^18^ and exhibited comparable overall scale and angular-energy behavior, including the pronounced forward high-energy component and its reduction with increasing emission angle (Supplementary Figure S4). This assessment was performed for the particle-normalized neutron source term before applying dose-defined workload normalization.

These trends can be interpreted in terms of the underlying spallation reaction mechanisms. At small emission angles in low-Z media such as water, intra-nuclear cascade processes contribute a forward-directed, high-energy neutron component whose magnitude increases with projectile mass^19^. As the target mass number and effective thickness increase, pre-equilibrium emission and evaporation processes become comparatively more significant, resulting in neutron spectra characterized by broader angular distributions and lower energies^19,20^. In metallic beam-loss targets such as copper, SUS304, and silicon steel, pre-equilibrium emission (20–100 MeV) and evaporation (approximately 1–20 MeV and 300 eV–1 MeV) account for a larger fraction of the spectrum, while attenuation in the high-density medium preferentially suppresses forward neutrons above 100 MeV. Consequently, the forward fraction decreases and the fluence shifts toward intermediate energies and larger angles, weakening the spectral contrast that distinguishes heavier ions from helium in water.

These spectral shifts have direct dosimetric consequences. Because neutron penetration depth increases with energy, dose at depth is governed primarily by the high-energy portion of the spectrum. Under metallic beam-loss target conditions, the forward fraction of neutrons above 100 MeV is reduced for all projectiles compared to the water target, but more strongly for the heavier ions than for helium. In the 0–10° bin, the carbon–helium difference in the forward fraction above 100 MeV decreases from 13.2% points in water to 4.98% points in copper, thereby reducing the carbon-to-helium neutron effective-dose ratio from 2.74 to 1.57. Given the similarity of the forward-cone spectral fractions among the metallic beam-loss targets (Cu, SUS304, and silicon steel), the same mechanism—compression of the inter-ion high-energy contrast in the forward cone—applies across these metallic beam-loss media. Accordingly, in metal-rich configurations, the relative contribution of helium to the neutron effective dose increases compared with water-based conditions.

Although the forward-cone spectral fractions in metallic beam-loss targets are broadly comparable (Fig. 7), the shielding-relevant dose under dose-based normalization is ultimately governed by the magnitude of forward neutron production, which can vary with target composition even among metallic media. Cu is a single-element target, whereas SUS304 and silicon steel are Fe-based alloys with different alloying constituents (Cr and Ni for SUS304, and Si for silicon steel), and such compositional differences can modulate thick-target neutron production through changes in nuclear reaction channels and secondary-particle cascades. Consistent with this interpretation, the dose-scaled forward neutron yield for helium exhibits a larger material dependence than for the heavier ions (Fig. 6): helium exceeds the carbon reference by 80% in Cu and increases further in SUS304 and silicon steel (93% and 97%), whereas oxygen and neon remain within 2% across the metallic beam-loss targets. Because the forward spectral structure within 0–10° remains broadly similar across the metallic beam-loss targets (Fig. 7), this material dependence is most plausibly attributed to differences in absolute neutron yield rather than to qualitative restructuring of the forward-cone energy composition. Accordingly, in practical terms, these results indicate that metallic beam-loss media should not be treated as a single equivalence class for helium under dose-defined workloads, and that Fe-based targets can represent more conservative local conditions than Cu even when their forward spectral fractions appear comparable.

Shielding evaluations should therefore be conducted with explicit consideration of the materials involved in beam loss and interaction, as material-dependent spectral characteristics govern neutron transport, attenuation, and the resulting dose outside the shielding. Such material-specific analyses provide more robust and conservative bases for shielding design, benchmarking, and safety-margin determination.

### Biological considerations for different ion species

Biological effectiveness further modulates the relative shielding contribution of different ion species when comparisons are made on an RBE-weighted dose basis. The biological effects of different ion species are commonly characterized by the RBE, which generally increases with LET. However, beyond approximately 100 to 200 keV/µm, RBE no longer increases and tends to saturate or decline due to the overkill effect, in which additional energy deposition no longer produces further biological gain^21^. In our analysis, the dose-mean lineal energy (y_D_) at the Bragg peak was 88.8 keV/µm for carbon and 205 keV/µm for neon, yet the corresponding RBEs were 3.43 and 3.74, respectively, showing only about a 10% difference. This indicates that despite substantial variation in LET, the effective RBE values among these ions converge near the clinically relevant region of maximum dose deposition.

We adopted the modified MKM because it is a clinical RBE model that accounts for overkill effect in high-LET region, which is relevant around the Bragg peak^22^. In the clinical carbon-ion dose system based on the modified MKM, RBE-weighted dose is defined using fixed HSG-based reference cell-survival parameters. Accordingly, the RBE-weighted dose used in the present study should be interpreted as a clinical dose convention for treatment-workload normalization, rather than as a tissue-specific biological endpoint. This is consistent with current clinical carbon-ion treatment planning, in which α/β values are not routinely varied for each tissue or disease site. Although the model has been most extensively validated for carbon-ion dose calculation, ongoing studies are extending and experimentally validating new models for multi-ion radiotherapy^23^. If clinically implemented RBE models incorporating cell- or tissue-specific α/β dependence, such as MCF-MKM-type approaches^24^, become available in the future, their impact on RBE-weighted shielding workload normalization could be evaluated using the same dose-based assessment approach. However, because major RBE models share a common structure characterized by LET-dependent increase and saturation at high LET, the relative ordering of ion species is expected to be preserved^25^. Consequently, the selection of the modified MKM does not alter the qualitative shielding conclusions, particularly the identification of carbon as the most robust conservative reference ion. Differences among RBE models may influence marginal cases, such as the local comparison between high-energy helium and carbon, but these effects remain within the combined model and systematic uncertainties.

The 2.0-cm integration window used in the pristine-peak analysis should be interpreted as a reference scoring interval for controlled inter-ion comparison, rather than as a representation of the full clinical target volume. The sensitivity analysis using window lengths from 1 to 6 cm showed that the particle-number ratios were stable for window lengths of 2.0 cm and larger. In addition, the SOBP-based normalization using 1-, 3-, and 5-cm modulation widths showed little dependence on SOBP width and preserved the same inter-ion trends. These results indicate that the particle-number normalization was not strongly dependent on the specific 2.0-cm reference interval and remained applicable under clinically extended dose-delivery conditions within the range examined.

### Scope and future applicability

This study compared RBE-weighted dose and neutron effective dose among different ion species under simplified geometric conditions. The shielding geometry was deliberately reduced in complexity to isolate ion-dependent effects, whereas actual heavy-ion therapy facilities involve more intricate structural layouts and heterogeneous shielding materials. High-density concrete is one practical strategy for reducing shielding thickness in carbon-ion therapy facilities; however, its shielding performance depends on both density and elemental composition, which can vary during construction. Shielding guidance notes that high-Z aggregates or scrap steel/iron can increase concrete density and effective atomic number, but also describes practical challenges related to pouring, handling, structural stability, and quality control, including possible nonuniformity caused by settling of high-Z aggregates^26^. In line with this practical concern, Wang et al. reported variability in density and iron content in a magnetite-based heavy concrete test block for carbon therapy and emphasized that density, high-Z content, and shielding thickness should be balanced in engineering design^27^. Such material variability may affect facility-specific shielding performance and reinforces the need for site-specific material verification.

In addition to high-density-concrete optimization, local high-Z reinforcement with steel or iron is also a practical shielding strategy. Particle-therapy shielding guidance supports the use of steel as a dense shielding material for high-energy neutrons, particularly when space is limited, and recommends placing hydrogenous or concrete shielding downstream of the steel layer to attenuate secondary lower-energy neutrons generated in steel^26^. Consistent with this shielding concept, the BULK-II shielding-design tool for proton and carbon particle-therapy accelerator facilities includes calculations behind iron, concrete, and combined iron–concrete shielding materials^28^. A practical example is provided by the Heidelberg Ion-Beam Therapy Center, where the radiation area is enclosed by reinforced-concrete walls and ceiling up to 2.5 m thick, with embedded 50-cm-thick steel plates or reinforced-concrete ribs^29^. Accordingly, we additionally examined an iron-fronted concrete configuration as a representative high-Z local-reinforcement case. This supplementary configuration reduced the absolute transmitted dose but did not substantially change the equilibrium-region TVL behavior or the relative trends among ion species. Therefore, when applying the present findings to practical facility design, further verification using site-specific geometry, concrete density, elemental composition, construction quality, and local shielding layout is recommended.

As an additional lateral shielding check, 90° scattered-neutron yields and effective doses were evaluated for representative H₂O–concrete and Cu–concrete configurations and compared with the corresponding 0° forward-directed values. The particle-normalized yield analysis showed that the relative lateral contribution did not contradict the carbon-based reference interpretation derived from the forward-direction assessment. In H₂O, the 90°/0° total-yield ratios were similar among carbon, oxygen, and neon, while helium showed a smaller ratio than carbon. In Cu, the 90°/0° total-yield ratio was highest for carbon among the tested ions. The 90°/0° yield ratio for neutrons with energies above 100 MeV was also highest for carbon in both target conditions. The effective-dose comparison further showed that the 90°/0° neutron effective-dose ratio at 50 cm concrete depth remained at 1% or lower for all tested ion and target conditions. These results indicate that the 90° lateral component did not introduce a shielding-relevant ion trend that would require revising the carbon-based reference evaluation (Supplementary Table S2).

Neutron dose was expressed in terms of effective dose using ICRP 116 conversion coefficients. Because the same coefficients were applied to all ion species, the relative inter-ion trends are expected to be robust with respect to the choice of protection quantity. For applications requiring operational quantities such as ambient dose equivalent H*(10), additional conversion or direct scoring of the corresponding quantity would be required.

Because the reported statistical uncertainties do not include model-related systematic uncertainties, absolute shield-transmitted neutron effective doses and TVLs should be interpreted as model-dependent estimates rather than exact shielding constants. However, the primary comparisons in this study were based on ion-to-carbon ratios under matched simulation conditions, so systematic components related to transport, dose conversion, scoring, and geometry may be partially cancelled in the ratios. This consideration is particularly relevant because carbon, oxygen, and neon were treated using the same JQMD/GEM framework in PHITS, whereas helium was treated using a different INCL/GEM model chain. Accordingly, the small He/C differences observed only for helium at 250 MeV/u under metallic beam-loss conditions, corresponding to approximately 4.8–14.6% relative to carbon, were interpreted as comparable shielding burdens rather than definitive ion-specific differences. In contrast, oxygen and neon remained below carbon at the representative depth of 200 cm, supporting the robustness of the lower shielding burden observed for these ions.

Although the shielding simulations used maximum-energy pristine beams as conservative reference cases, the dose-normalization component was additionally evaluated using SOBP-based RBE-weighted dose integration to reflect clinically extended dose delivery. This distinction is appropriate because the highest-energy component governs deep-penetrating secondary neutrons, whereas particle-number-per-dose normalization should reflect the treatment dose-delivery condition. Under the conditions examined, the SOBP-based normalization did not reduce the conservativeness of the carbon-based shielding assessment.

This SOBP analysis was limited to the water-phantom dose-normalization step and was not intended to represent patient-specific treatment plans or facility-specific beam-loss patterns. In addition, the beam energies considered correspond to the current clinical maximum for helium ions and to the highest acceleration energies available for oxygen and neon in existing heavy-ion synchrotrons. These conditions reflect present technical limits; however, if future accelerator developments enable higher beam energies or extended treatment ranges for oxygen and neon, shielding characteristics should be re-evaluated to preserve the applicability of the present conclusions.

Despite these simplifications, the assumptions were introduced to ensure consistency and conservativeness in inter-ion comparisons and are not expected to substantially alter the relative trends or the principal conclusions derived under the RBE-based normalization.

## Conclusion

This study examined neutron shielding characteristics for multi-ion heavy-ion therapy from the perspective of how shielding workloads should be defined in facilities employing multiple ion species. Shielding effectiveness was evaluated using dose-defined workloads that reflect actual facility operation, with treatment-related workload defined in terms of RBE-weighted dose and quality assurance (QA) workload defined in terms of physical dose.

The results demonstrate that conclusions regarding conservative shielding conditions depend explicitly on the dose quantity used for normalization. Under normalization to unit RBE-weighted dose, representing clinical treatment workload, carbon ions generally produce the largest neutron effective dose among the ions considered, while helium ions may approach or exceed the carbon-based contribution under specific combinations of beam energy and material. In contrast, when shielding effectiveness is evaluated per unit physical dose, corresponding to QA and commissioning activities, carbon remains the most conservative reference ion across all conditions examined, and helium does not exceed the carbon-based shielding contribution.

By distinguishing between treatment and QA workloads and by formulating shielding assessment in terms of dose-based quantities rather than particle fluence, the present study establishes a coherent and operationally realistic basis for multi-ion shielding evaluation. This approach resolves ambiguities inherent in particle-based comparisons and enables conservative yet practical shielding design. These findings support the continued use of carbon as the primary reference ion for both treatment- and QA-related shielding assessment, highlighting the need to account for high-energy helium under specific RBE-weighted, metallic beam-loss scenarios in which its shielding contribution can approach the carbon reference. This distinction provides a transparent basis for extending existing carbon-based shielding designs to multi-ion operation without compromising safety or introducing unnecessary conservatism.

## Supplementary Information

Below is the link to the electronic supplementary material.

Supplementary Material 1

## Data Availability

The datasets generated during and/or analysed during the current study are available from the corresponding author on reasonable request.
