# Supplementary material for: Shielding assessment for multi-ion radiotherapy based on ion-specific dose-defined workloads
Source: Sci Rep. 2026 Jun 19;16:28153. doi: 10.1038/s41598-026-59143-0 (PMC13554122; doi:10.1038/s41598-026-59143-0)
Supplement: Supplementary file 1 [file 41598_2026_59143_MOESM1_ESM.docx]

Supplementary Figure S1. Simulation geometry with 50 cm iron shielding. Depth x in the shielding wall is calculated from the upstream surface of the iron wall (x=0)

Supplementary Figure S2. Depth-dependent neutron effective dose per unit physical dose for helium, carbon, oxygen, and neon beams across (a) water target and (b) copper target with 50 cm iron and 4.5 m concrete shielding configurations.

Supplementary Figure S3. Depth-dependent neutron effective dose per unit RBE-weighted dose for helium, carbon, oxygen, and neon beams across (a) water target and (b) copper target with 50 cm iron and 4.5 m concrete shielding configurations.

Supplementary Figure S4. Particle-normalized angular-energy neutron source term for the 430 MeV/u carbon-on-water condition. The PHITS T-cross fluence spectra were converted to double-differential neutron yields, and plotted in the same format as Satoh et al.; spectra at 30°, 45°, 60°, 75°, and 90° were multiplied by 10^−1^, 10^−2^, 10^−3^, 10^−4^, and 10^−5^, respectively, for visual separation.

Supplementary Table S1. Effective dose ratios between ISO and AP fluence-to-effective-dose conversion geometries in the representative Cu–concrete configuration. The ISO/AP ratio was calculated by dividing the neutron effective dose obtained using ISO conversion coefficients by that obtained using AP conversion coefficients at the same concrete depth.

| Ion species | Effective dose ratio (ISO/AP) | | |
| --- | --- | --- | --- |
|  | **50cm** | **100cm** | **200cm** |
| He-4 | 79% | 76% | 76% |
| C-12 | 78% | 78% | 77% |
| O-16 | 79% | 79% | 79% |
| Ne-20 | 79% | 80% | 77% |

Supplementary Table S2. Lateral-to-forward neutron-yield and effective-dose ratios for representative H₂O–concrete and Cu–concrete configurations. The neutron-yield ratios were calculated by dividing the 90° lateral yield by the corresponding 0° forward yield. The 90° lateral yield was evaluated using the combined 80–100° angular bin. The high-energy yield ratio represents neutrons with energies above 100 MeV. The effective-dose ratio was evaluated at 50 cm concrete depth.

| Target | Ion | Neutron yield ratio  (90°/0°) | | Effective dose ratio (90°/0°) |
| --- | --- | --- | --- | --- |
|  |  | **Total** | **> 100 MeV** | **50cm** |
| Water | He-4 | 5.5% | 0.1% | 1.0% |
|  | C-12 | 6.3% | 0.3% | 0.7% |
|  | O-16 | 6.3% | 0.2% | 0.2% |
|  | Ne-20 | 6.2% | 0.2% | 0.2% |
| Cu | He-4 | 32.5% | 0.3% | 1.0% |
|  | C-12 | 41.2% | 0.7% | 1.0% |
|  | O-16 | 38.2% | 0.6% | 0.3% |
|  | Ne-20 | 35.5% | 0.5% | 0.2% |
